# Disproportionate Cybersexual Victimization of Women from Adolescence into Midlife in Spain: Implications for Targeted Protection and Prevention

**DOI:** 10.3390/bs15111571

**Published:** 2025-11-17

**Authors:** Carlos J. Mármol, Aurelio Luna, Isabel Legaz

**Affiliations:** Department of Legal and Forensic Medicine, Biomedical Research Institute of Murcia (IMIB), Regional Campus of International Excellence “Campus Mare Nostrum”, Faculty of Medicine, University of Murcia (UMU), 30100 Murcia, Spain; cj.marmol@um.es (C.J.M.); aurluna@um.es (A.L.)

**Keywords:** adolescence, corruption of minors, cybersexual victimization, grooming, sex differences, sexual abuse, sexual harassment, Spain

## Abstract

Cybersexual victimization is a growing public health concern with severe psychosocial consequences, particularly for younger populations. Despite growing awareness of its prevalence, understanding how cybersexual victimization evolves across different demographic and regional contexts remains limited. The aim was to analyze sex- and age-specific temporal trends and projections of cybersexual victimization in Spain (2011–2022), disaggregated by sex, age group, autonomous community, and offense type, to identify where disparities emerge and persist (particularly from adolescence (<18) into midlife) while also examining gender and regional inequalities to provide evidence for prevention strategies that are both gender-sensitive and tailored to different developmental stages and territorial contexts. Spanish national police-reported data on seven cybersexual offenses (sexual abuse, sexual harassment, corruption of minors, grooming, exhibitionism, child sexual abuse images, and sexual provocation) from 2011 to 2022 were analyzed. Data were disaggregated by sex, age group, and regions. Mean rates per 100,000 inhabitants were calculated, independent-sample *t*-tests assessed sex differences, and linear regression models projected trends to 2035 for each age-sex group. Between 2011 and 2022, cybersexual crimes in Spain increased across most offense types, with grooming, child sexual abuse images, and contact offenses showing the steepest upward trends (all *p* < 0.001). Women consistently presented higher mean victimization rates than men in most offense types and age groups. Among those under 18, mean grooming rates were 2.55 for females versus 0.95 per 100,000 for males (*p* < 0.001), with significant differences also in corruption of minors (*p* < 0.01). In young adulthood (18–25 years), women showed higher rates in sexual harassment (*p* < 0.001) and sexual abuse (*p* < 0.01), while, in midlife (26–40 and 41–50 years), female predominance persisted for sexual harassment, sexual abuse, and sexual provocation (all *p* < 0.05). Projections to 2035 indicate that sex gaps will remain or widen, particularly among females under 18 and in the 26–40 age group. The Balearic, Canary Islands, and Andalusia regions recorded the highest mean rates, whereas Galicia and Castilla-La Mancha reported the lowest. Cybersexual victimization in Spain disproportionately affects females from adolescence into midlife, with the most considerable disparities emerging before age 18 and persisting into adulthood. The combination of rapid offense growth, persistent sex-based disparities, and marked regional inequalities underscores the urgent need for gender-sensitive, developmentally targeted prevention strategies that address both early vulnerability and the reinforcement of risk in adult digital environments.

## 1. Introduction

In today’s socio-digital landscape, daily interactions across daily life are increasingly mediated by online platforms and connected devices (Hampton, 2016; Kostin et al., 2021; Relinque-Medina & Álvarez-Pérez, 2024). While digitalization has brought notable benefits for communication and access to information, it has also facilitated new and complex forms of sexual and gender-based violence that are difficult to detect, regulate, and prevent (Grabosky & Smith, 2018; Stratton et al., 2014; Trittin-Ulbrich et al., 2021).

The widespread use of social media, messaging applications, and image-sharing platforms has blurred the boundaries between public and private spaces, allowing the violation of sexual autonomy and privacy to occur through digital channels. Within this context, the concept of cybersexual victimization has emerged to describe behaviors in which technology becomes an instrument of sexual coercion, humiliation, or exploitation (Henry & Powell, 2016; Jurasz & Barker, 2021).

Cybersexual crimes encompass a broad spectrum of offenses that violate sexual freedom and integrity through digital means. According to the typology used by the Spanish Ministry of the Interior, these include sexual abuse, sexual harassment, corruption of minors, grooming, exhibitionism, dissemination of child sexual abuse images, and sexual provocation (Datos—Portal Estadístico, n.d.). Sexual abuse in digital settings refers to unwanted sexual acts, exposure to explicit content, or coercive interactions conducted through electronic media. Sexual harassment involves repetitive, intrusive, or degrading messages of a sexual nature that create an atmosphere of intimidation or humiliation. Corruption of minors includes exposing children or adolescents to pornographic content or inducing them to participate in sexualized behaviors, while grooming describes the gradual process by which an adult gains a minor’s trust to obtain sexual contact, images, or personal information (de Santisteban & Gámez-Guadix, 2018; Whittle et al., 2013).

Exhibitionism refers to the deliberate exposure of sexual organs or acts through digital platforms, frequently directed at unwilling recipients, and sexual provocation encompasses behaviors that incite or pressure others, particularly minors, into producing or engaging with sexual content. Finally, the dissemination of child sexual abuse images represents one of the most severe forms of exploitation, as it perpetuates harm and revictimization over time (Endrass et al., 2009). Together, these behaviors illustrate how digital tools extend the reach of sexual violence into private and domestic spaces, amplifying both its frequency and psychological impact (Fatima, 2024; Henry et al., 2020).

These offenses are not isolated events but expressions of broader social and structural inequalities that shape who is most at risk and how violence manifests. Research has shown that cybersexual victimization is strongly influenced by gender, age, socioeconomic status, and digital literacy (Trittin-Ulbrich et al., 2021). Adolescent girls and young women are consistently identified as the most vulnerable groups, often facing sustained exposure to online harassment, sexual coercion, and non-consensual image sharing (Finkelhor et al., 2022; Martínez-Bacaicoa et al., 2024). Such experiences may produce lasting psychological consequences—such as anxiety, depression, and social withdrawal—and are often accompanied by a normalization of sexualized aggression and reduced empathy, fostered by online anonymity and exposure to explicit content (Guggisberg, 2020; Lim et al., 2017).

Emerging technologies, including deepfake pornography, encrypted communications, and artificial intelligence-generated sexual material, further complicate detection and accountability (Alanazi et al., 2024; Lin, 2025; Mohamed et al., 2023). 

These digital innovations intersect with pre-existing gendered power imbalances and contribute to the desensitization of sexual violence in online spaces, thereby reinforcing women’s and girls’ vulnerability (Bernstein et al., 2022). Moreover, digital tools have become instruments of coercion within intimate relationships, extending beyond adolescence into adulthood and midlife, suggesting that cybersexual victimization follows a life-course pattern rather than being confined to youth (Rogers et al., 2022).

In Spain, the study of cybersexual violence has acquired growing relevance. National survey has revealed that over 60% of adolescents have experienced some form of online sexual aggression, with unsolicited sexual content and harassment being the most frequent behaviors (Calderón Gómez et al., 2024; Montiel et al., 2016).

These risks are unevenly distributed across regions, with higher incidence rates in territories characterized by lower educational attainment and socioeconomic vulnerability, such as Andalusia, Extremadura, and Murcia (Espinosa Zárate et al., 2023). This regional variability underscores how structural and cultural asymmetries influence exposure to digital risks and access to protection systems (Thompson, 2020). Spanish institutions have advanced prevention and awareness through programs such as Internet Segura for Kids (IS4K), coordinated by the National Cybersecurity Institute (INCIBE) (Internet Segura for Kids (IS4K) | INCIBE, n.d.). Nevertheless, there remains a lack of longitudinal research examining the evolution of cybersexual victimization disaggregated by sex, age.

This study contributes to current knowledge on technology-facilitated sexual violence by presenting one of the first longitudinal and regionally disaggregated analyses of cybersexual victimization in Spain. By examining trends by sex, age group, and autonomous community over twelve years, it offers a detailed view of how digital sexual violence develops across different life stages and social contexts. The findings provide a solid empirical basis for future research and for the design of prevention strategies that address gender and regional inequalities in online victimization.

The aim was to analyze sex- and age-specific temporal trends and projections of cybersexual victimization in Spain (2011–2022), disaggregated by sex, age group, autonomous community, and offense type, to identify where disparities emerge and persist, particularly from adolescence (<18) into midlife, while also examining gender and regional inequalities to provide evidence for prevention strategies that are both gender-sensitive and tailored to different developmental stages and territorial contexts.

## 2. Methods

### 2.1. Data Sources and Contextual Variables on Cyber-Sexual Crimes

Data for the present analysis were sourced from the Statistical Crime Portal (PEC), which contains official records of cyber-sexual crimes registered in Spain between 2011 and 2022 (Datos—Portal Estadístico, n.d.). The data are compiled annually by national, regional, and local law enforcement agencies. However, it should be noted that the Mossos d’Esquadra (Catalonia) and the Ertzaintza (Basque Country) began reporting arrest and investigation figures only from 2015 onward, which may result in incomplete records for these regions in earlier years. For regional comparisons, incidence rates were standardized per 100,000 inhabitants based on annual population estimates from the National Statistics Institute (INE) (Instituto Nacional de Estadística, n.d.). Rates per 100,000 inhabitants were calculated separately for males and females using population denominators disaggregated by sex and age, obtained from the Spanish National Statistics Institute (INE), to ensure correspondence with the categories of reported cases provided by the Ministry of the Interior. Data from Catalonia and the Basque Country before 2015 were excluded from regional comparisons for consistency. Regional data corresponds to where the victim filed the complaint with the police. No data imputation was performed.

### 2.2. Typology of Cyber-Sexual Offenses and Age Criteria for Victim Classification

The study encompassed various categories of cyber-sexual offenses, including exhibitionism, sexual provocation, harassment, abuse, corruption of minors or individuals with disabilities, child sexual abuse images, and digital grooming, understood as online interactions with minors pursued for sexual purposes. All incidents involving victims aged 0 to 17 were classified as child victimization. Age and sex disaggregation followed the classification system used in the official datasets, which grouped victims into the following age ranges: 0–17, 18–25, 26–40, 41–50, and 51 years and older. Analyses were conducted separately for male and female victims in each group.

### 2.3. Temporal Trend Analysis by Type of Cyber-Sexual Offense

Temporal trends in cyber-sexual crime incidence were analyzed through simple linear regression models. An initial model was applied to the aggregated national rate of reported all recorded cyber-sexual offenses from 2011 to 2022, using the standardized annual rate as the dependent variable and treating the calendar year as a continuous predictor. The slope coefficient (β) reflected the mean yearly change, while the coefficient of determination (R^2^) and the associated *p*-value assessed the model’s explanatory strength and statistical significance. Separate regression models were also run for each of the seven offense categories to estimate their growth trajectories over time. This approach was designed as a descriptive trend analysis, aimed at quantifying the rate and direction of change rather than inferring causality. The simple linear regression model was selected to provide a comparable and transparent measure of yearly change across heterogeneous offense types and demographic strata.

### 2.4. Projection of Trends and Predictive Modeling of Cyber-Sexual Crime Rates

A projection was carried out using annual time series data on sexual offense rates from 2011 to 2022. Linear regressions were employed to estimate the potential continuation of these offenses through 2035. Potential continuation was based on simple linear regression models fitted to annual incidence rates from 2011 to 2022. This approach was chosen to estimate medium-term trends clearly, focusing on general patterns rather than short-term fluctuations. For each sex (women/men) and age group (<18, 18–25, 26–40, 41–50, 51–65, >65), a linear trend model was fitted using ordinary least squares (OLS), with year as a continuous predictor. Model fit criteria (slope *p*-values, adjusted R^2^, residual analysis) were assessed to validate the consistency of the fit in each category. The projection was extended to 2035, considering a medium-term horizon, and 95% confidence intervals were constructed to represent the uncertainty associated with the forecast. It should be emphasized that these projections were not intended as predictive or inferential forecasts but as descriptive visual extrapolations of observed trends. The linear regression model was selected to ensure methodological consistency and comparability across the different offense types and demographic groups. This approach aimed to provide an interpretable overview of trend direction, rate of change, and persistence of disparities, with 95% confidence intervals included to represent the uncertainty associated with each extrapolation.

### 2.5. Statistical Analysis

Data were compiled into a structured database using Microsoft Access 11.0 (Microsoft Corporation, Seattle, WA, USA). Statistical analyses were conducted with SPSS version 29.0 (IBM Corp., Armonk, NY, USA). Results were reported as absolute frequencies (n), percentages, and incidence rates per 100,000 population. A one-way analysis of variance (ANOVA) was performed to assess regional differences in the mean rates of cyber-sex crime across Spain’s autonomous communities a one-way analysis of variance (ANOVA) was performed. Then, a post hoc Tukey’s Honestly Significant Difference (HSD) test was applied to conduct pairwise comparisons between all regions and minimize the risk of Type I errors. To assess whether the observed differences in victimization rates between male and female minors were statistically significant, a series of independent-sample *t*-tests was conducted for each offense type.

Additionally, absolute differences between mean victimization rates for women and men were calculated for each offense type and age group. These differences were graphically represented to illustrate the magnitude of sex disparities across the study period and in the projections. Figures were generated using GraphPad Prism version 5.04 (GraphPad Software, San Diego, CA, USA). Maps of the Spanish Autonomous Communities (CCAA) were created using Microsoft Excel (Office 2016), following the ISO 3166 standard established by the International Organization for Standardization (2020). Color intervals were manually defined to reflect meaningful variations in regional crime rates across offense types.

## 3. Results

### 3.1. Annual Evolution of Sexual Crime Rates by Region in Spain

**Figure 1:**
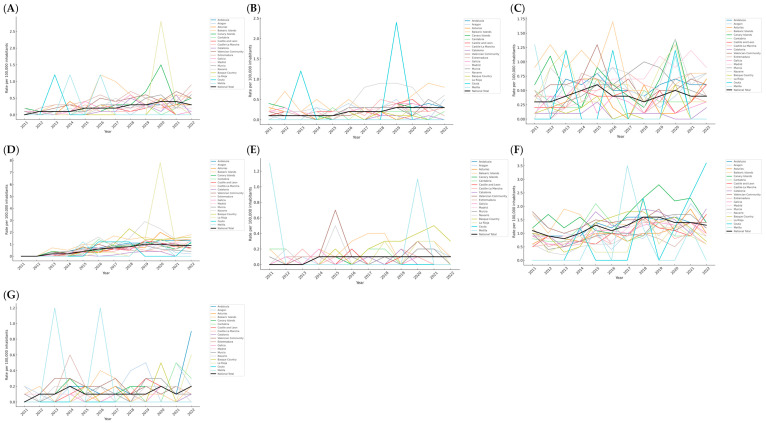
Annual evolution of cybersexual crime rates by region in Spain (2011–2022). Trends are shown for seven offense categories: (**A**) sexual abuse, (**B**) sexual harassment, (**C**) corruption of minors, (**D**) grooming, (**E**) exhibitionism, (**F**) child sexual abuse images, and (**G**) sexual provocation. Rates are expressed per 100,000 inhabitants.

Between 2011 and 2022, the national rate of reported sexual abuse increased from 0.0 to 0.4 cases per 100,000 inhabitants in 2020 and 2021, then declined slightly to 0.3 in 2022 (Figure 1A). Andalusia showed the highest peak, with 2.8 in 2020, followed by Madrid, which showed 1.47 in the same year. These values contrast with much lower and more stable rates observed in the Basque Country, Cantabria, and La Rioja, which generally remain below 0.4. Other regions, such as the Balearic Islands, Melilla, and the Valencian Community, exceeded 1.0 in specific years. Regional variability was notable, with some communities consistently above the national mean and others well below.

Regarding sexual harassment, the national rate increased gradually from 0.1 in 2011 to 0.3 in 2019 onward (Figure 1B). The Balearic Islands recorded the highest value, reaching 0.9 in 2021, while the Canary Islands followed with 0.6 in 2022. Navarra experienced a rise from 2017 to 2019, also peaking at 0.9. Ceuta showed a sharp and isolated increase to 2.4 in 2019. In contrast, Catalonia, the Basque Country, La Rioja, and Melilla reported very low or null values throughout the period. Madrid, Murcia, and Galicia started at 0.2 and gradually increased to between 0.3 and 0.5, reflecting a more moderate but sustained evolution. Regarding the corruption of minors, the national rate fluctuated between 0.3 and 0.6, settling at 0.4 in recent years (Figure 1C). The Balearic Islands consistently recorded some of the highest rates, with a maximum of 1.7 in 2016. Madrid reached 1.2 in 2021, while Navarra peaked at 1.4 in 2020. La Rioja had elevated rates in 2013 and 2020, at 1.2 and 1.3, respectively. Murcia showed a progressive increase from 0.0 in 2011 to 1.0 in 2018. Meanwhile, Catalonia, the Basque Country, and Melilla (except in 2011) remained close to zero. The Canary Islands and Aragón also presented intermittent peaks, especially in 2012 and 2016. For contact-related crimes involving minors under 16, the national rate rose from 0.0 in 2011 to 0.9 in 2022 (Figure 1D). The Balearic Islands surpassed the national figure in several years, reaching 1.8 in 2022. Asturias recorded 2.0 in 2020, and both Navarra and the Basque Country showed pronounced peaks, reaching 2.9 in 2019 and 2.3 in 2018, respectively. La Rioja registered a notable spike of 7.8 in 2020, which stands out compared to the rest of the regions. Murcia and Andalusia surpassed 1.0 by 2018 and 2020, respectively, while Catalonia consistently reported rates below 0.4. Exhibitionism remained low nationwide, between 0.0 and 0.1 (Figure 1E). However, the Balearic Islands recorded higher values in multiple years, reaching 0.4 in 2017 and 2018. Navarra and the Basque Country peaked at 0.5 in 2015 and 2021, respectively. La Rioja reported 0.3 in 2018 and 2020. Melilla stood out for isolated peaks of 1.3 in 2011 and 1.1 in 2020. In contrast, Ceuta, Castilla y León, and Catalonia reported almost no variation, remaining consistently below or at national levels. The national rate of reported child sexual abuse image offenses rose from 1.1 to 1.3 between 2011 and 2022, peaking at 1.6 in 2018 and 2019 (Figure 1F). The Canary Islands consistently reported the highest figures, with a maximum of 2.8 in 2019. The Valencian Community, Catalonia, the Basque Country, and Andalusia also maintained elevated rates. Ceuta and Melilla recorded single-year spikes of 3.6 in 2022 and 3.5 in 2017, respectively. Aragón, Cantabria, and Castilla y Leon occasionally increased, while Navarra, Galicia, and Extremadura showed more moderate and stable values. Finally, sexual provocation remained the least reported category, with national rates ranging from 0.0 to 0.2 (Figure 1G). Andalusia registered the highest value, with 0.9 in 2022. Aragón and Cantabria reached 0.5 in 2018, 2019, and 2021. La Rioja increased to 0.6 in 2022 after several years of no recorded cases. Ceuta reported no cases throughout the period, while Melilla had isolated peaks of 1.2 in 2013 and 2016.

### 3.2. Regional Deviations from National Means by Offense Type

**Figure 2:**
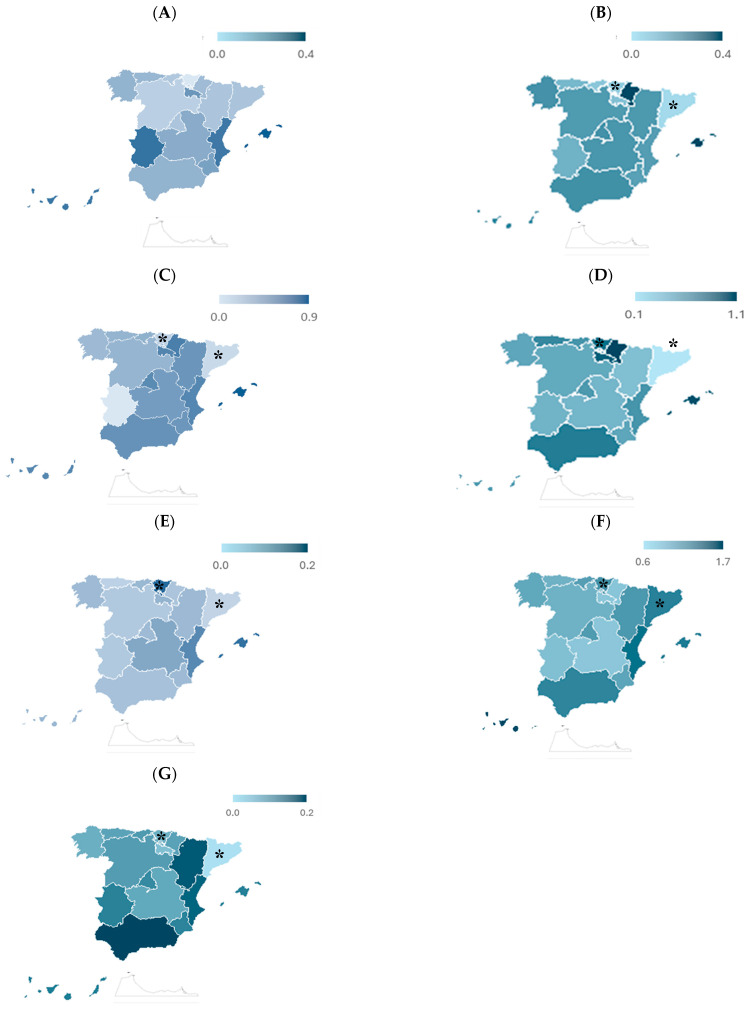
Mean cybersexual crime rates by region and offense type (2011–2022). Mean rates per 100,000 inhabitants are displayed for each autonomous community and compared to the national mean. Offense categories include: (**A**) sexual abuse, (**B**) sexual harassment, (**C**) corruption of minors, (**D**) grooming, (**E**) exhibitionism, (**F**) child sexual abuse images, and (**G**) sexual provocation. * Data for Catalonia and the Basque Country before 2015 are incomplete due to partial reporting and should be interpreted cautiously.

Figure 2 shows a comparative view of the means of cyber-sex crimes across Spain’s autonomous communities between 2011 and 2022, broken down by offense type. This regional perspective helps identify areas that have remained systematically above or below the national mean in specific categories over the years. In the case of sexual abuse (Figure 1A), the national stand stood at 0.2 cases per 100,000 inhabitants. While most communities hovered near this figure, some recorded consistently higher means, such as the Balearic Islands, the Canary Islands, the Valencian Community, and Extremadura. Conversely, Cantabria, Castile, León, and the Basque Country reported lower rates throughout the period. Sexual harassment (Figure 2B) followed a similar pattern. The national mean was again 0.2, but the Balearic Islands and Navarre reported higher mean rates (0.4), followed closely by the Canary Islands and Ceuta (0.3).

In contrast, Catalonia and Melilla reported little to no cases on average, while other regions like Asturias, La Rioja, and the Basque Country remained below the national figure. As for the corruption of minors (Figure 2C), the mean rate nationwide was 0.4. The Balearic Islands again peaked at 0.9, followed by Navarre (0.7) and a cluster of communities (including the Canary Islands, the Valencian Community, and Madrid) with means around 0.6. By comparison, Asturias, Castile and León, Galicia, and Ceuta reported lower rates (0.3), while Catalonia and the Basque Country showed the lowest incidence, with values around 0.1. Grooming or online contact with minors (Figure 2D) displayed greater variation between regions. The national mean was 0.6, but in Navarre, it reached 1.1, and in the Balearic Islands and La Rioja, it exceeded 0.9.

In contrast, Melilla (0.3) and Catalonia (0.1) remained well below the national level. Exhibitionism (Figure 2E) was the least frequent offense on mean, with a national rate of 0.1. Most regions reported values near that mark. The Balearic Islands, the Basque Country, and Melilla were the only exceptions, with slightly higher means of 0.2. In contrast, Asturias, Castile and León, Catalonia, Extremadura, and Ceuta reported few cases. Child sexual abuse images (Figure 2F) showed the highest overall mean among all the cyber-sex crimes analyzed, with a national rate of 1.2. The Canary Islands led this category (1.7), followed by the Valencian Community (1.4), Andalusia, the Balearic Islands, and Catalonia (1.3). Despite occasional spikes in specific years, Melilla had the lowest mean (0.6). Several other communities, such as Castilla-La Mancha, Extremadura, and Navarre, fell below the national means.

Finally, sexual provocation (Figure 2G) was relatively uncommon, with a stable national mean of 0.1. Most regions followed this trend. Only a few, like Andalusia, Aragón, the Valencian Community, and Melilla, recorded slightly higher values (0.2). Catalonia and Ceuta did not report any cases during the study period.

### 3.3. Projected Trends of National Cyber-Sexual Crime Rates in Spain (2011–2035)

**Figure 3:**
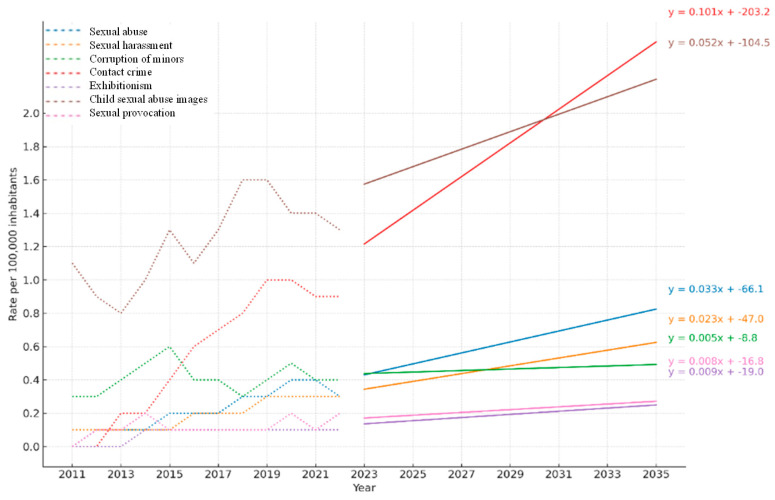
Projected trends in national cybersexual crime rates in Spain by offense type (2011–2035). Annual rates per 100,000 inhabitants are shown for 2011–2022, with linear projections to 2035. Offense types include sexual abuse, sexual harassment, corruption of minors, contact crime, exhibitionism, child sexual abuse images, and sexual provocation. Dashed lines represent observed data; Solid lines represent projected trends based on linear regression equations (shown in the figure).

The projection analysis of national sexual crime rates in Spain from 2011 to 2035 revealed distinct trends by crime type (Figure 3). First, pornography was observed to have the highest rates throughout the entire period analyzed. In 2011, the rate was 1.1 per 100,000 inhabitants, and, although it experienced slight fluctuations, it remained high, reaching 1.3 in 2022. Its linear projection suggests a slightly upward trend, remaining the most frequent sexual crime in relative terms over the coming years. On the other hand, contact crimes showed a very pronounced growth pattern. Starting from a zero rate in 2011, they increased to 0.9 per 100,000 inhabitants in 2022. This crime registered one of the steepest slopes, anticipating a sustained rise that could place the rate above 1.5 before 2035 if the trend continues.

On the other hand, both sexual abuse and sexual harassment exhibited a stable but increasing trend. Their projection equations indicate slight but continued growth, projecting rates around 0.5 to 0.6 by 2035. The analysis of minors’ corruption showed a more variable trend. After an initial increase with a rate of 0.6 in 2015, they stabilized at 0.4 starting in 2016. Their projection revealed an almost flat trend, with a slight upward slope that suggests continuity in their current behavior.

In contrast, the crimes of indecent exposure and sexual provocation remained at very low levels throughout the period. Both crimes hovered around 0.1 per 100,000 inhabitants, showing no signs of significant change. Projections confirm this stability, with no significant growth expected until 2035.

### 3.4. Evolution of Sexual Offense Rates in Spain by Age and Sex

**Figure 4:**
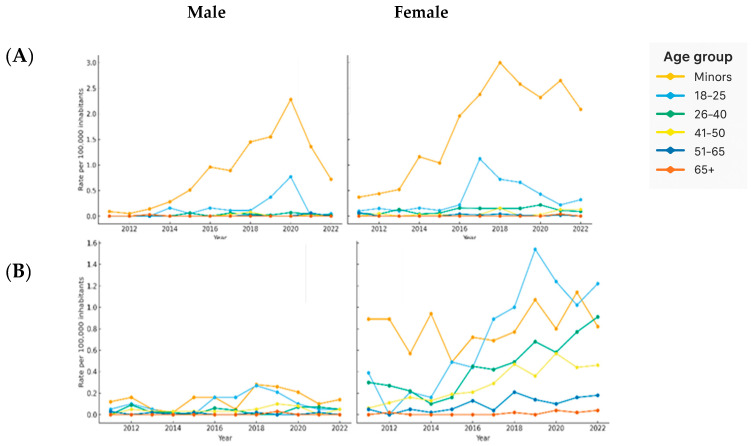

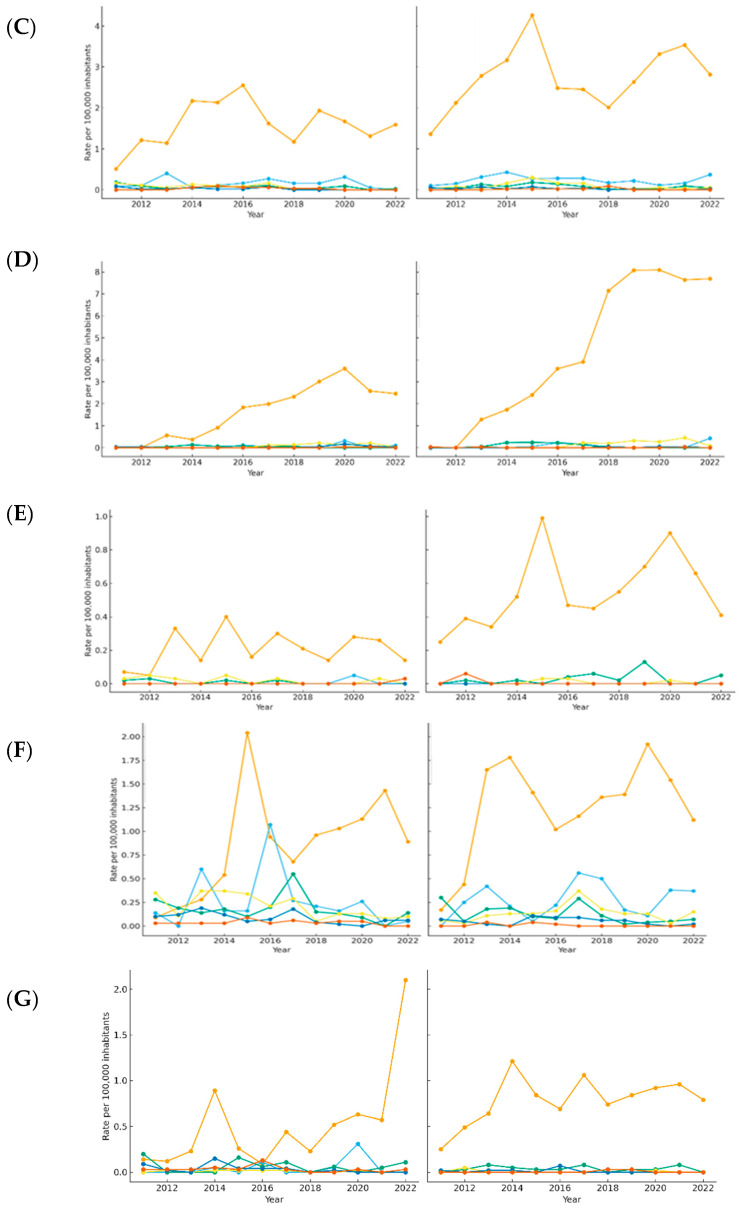
Annual trends (2011–2022) of cybersexual victimization rates in Spain by age group and sex. Each panel displays the evolution of police-reported victimization rates per 100,000 inhabitants, disaggregated by sex (male: left, female: right) and age group. The panels represent the following offense types: (**A**) sexual abuse, (**B**) sexual harassment, (**C**) corruption of minors, (**D**) grooming, (**E**) exhibitionism, (**F**) child sexual abuse images, and (**G**) sexual provocation.

The analysis of cybersexual victimization rates in Spain between 2011 and 2022 revealed a consistent predominance of female victims, particularly minors and young adult women, across all categories of offenses (Figure 4). In the case of sexual abuse (Figure 4A), it is noteworthy that victimization rates among female minors rose significantly from 0.37 cases per 100,000 inhabitants in 2011 to a peak of 3.00 in 2018, before slightly declining to 2.09 in 2022. Among male minors, the rate remained significantly lower, reaching only 1.36 in 2021 and falling to 0.72 in 2022. The mean victimization rate among female minors was 1.71 per 100,000 inhabitants, almost double that of male minors (0.86). Rates among females were higher across all age groups. For example, in the 18 to 25 age range, women recorded a mean of 0.36 compared to 0.15 for men, and in the 26 to 40 group, mean rates were 0.11 for women and 0.03 for men. Sexual harassment (Figure 4B) followed a similar trend, with female minors experiencing an increase from 0.89 in 2011 to a maximum of 1.14 in 2021, while male minors reported much lower rates, ranging from 0.02 to 0.28 throughout the period. Female minors had a mean rate of 0.81, significantly higher than the 0.14 recorded for male minors. This gender gap persisted across all age groups, with women aged 26 to 40 reaching 0.44 compared to 0.03 for men, and women aged 41 to 50 showing 0.29 versus 0.11 for their male counterparts. In the case of corruption of minors or persons with disabilities (Figure 4C), female minors consistently showed higher rates, starting at 1.36 in 2011 and peaking at 4.26 in 2015. The rate remained elevated in the following years, reaching 3.53 in 2021 and slightly decreasing to 2.81 in 2022. Male minors showed lower values, with rates fluctuating between 0.51 and 2.55, ending at 1.59 in 2022. Again, female minors recorded the highest mean victimization rate (2.74), compared to 1.58 for male minors. Although this gender disparity was most pronounced among minors, women also surpassed men in other age groups. For example, women aged 18 to 25 recorded a mean of 0.24 compared to 0.15 for men. The crime of sexual solicitation or grooming (Figure 4D) showed the most pronounced growth among all offenses. Rates for female minors sharply increased from 0.00 in 2011 to 7.69 in 2022, with the steepest rises occurring between 2015 and 2020. Male minors also experienced a notable increase, although at lower levels, reaching 2.46 in 2022. Female minors showed the highest overall mean rate, at 4.30, more than twice that of male minors (1.64). This upward trend extended into young adulthood, with mean rates of 0.08 for women aged 18 to 25, compared to 0.05 for their male counterparts.

In the case of exhibitionism (Figure 4E), rates for female minors remained below 1.00 throughout the period, with a modest increase from 0.25 in 2011 to 0.41 in 2022, although peaking at 0.99 in 2015. Among male minors, rates were generally lower, ranging from 0.05 to 0.33. Female minors continued to show a slightly higher mean victimization rate (0.55) compared to male minors (0.21). In older age groups, the differences remained modest but persistent, with women generally showing slightly higher exposure. Victimization through child sexual abuse images or child pornography (Figure 4F) among female minors increased from 0.17 in 2011 to 1.12 in 2022, with peaks observed in 2013 (1.65) and 2014 (1.78). Male minors showed slightly lower values, peaking at 2.04 in 2016 and recording 0.89 in 2022. Female minors had a mean of 1.25, while male minors showed a mean of 0.85. However, from age 26 onward, men showed higher mean rates than women.

Finally, the crime of sexual provocation (Figure 4G) also showed a predominant pattern of female victimization. Female minors experienced a progressive increase from 0.25 in 2011 to a peak of 1.06 in 2017, remaining above 0.90 in subsequent years. Male minors maintained lower rates, ranging from 0.12 to 0.89, although they displayed an exceptional peak in 2022 (2.10). Female minors recorded a mean rate of 0.79, compared to 0.52 for male minors. In all other age groups, women consistently showed greater exposure.

### 3.5. Sex-Based Differences in Cybersexual Victimization Rates Across Age Groups

**Figure 5:**
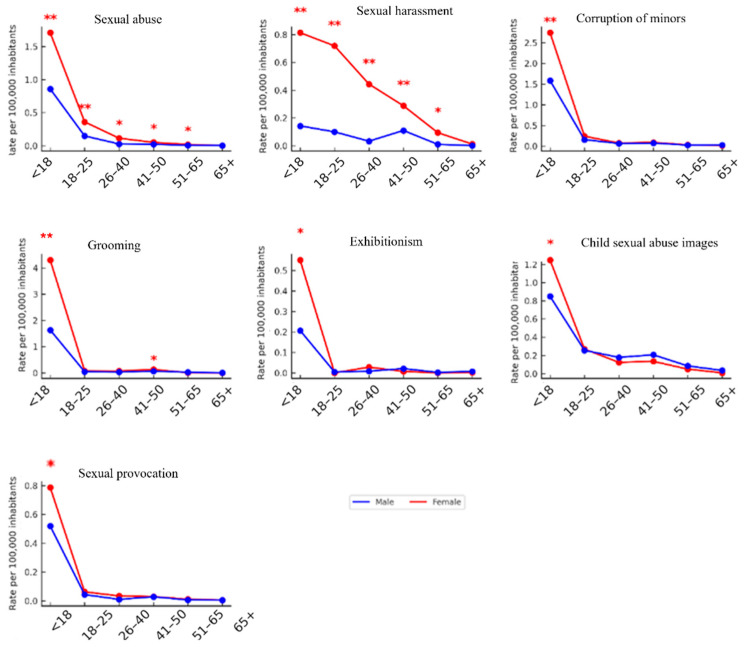
Age-specific rates of sexual offense victimization in Spain (2011–2022), by sex and type of offense. Mean annual rates per 100,000 inhabitants are shown for women (red) and men (blue) across six age groups. Each panel corresponds to a different offense category: sexual abuse, sexual harassment, child corruption, technology-facilitated contact offense, exhibitionism, child pornography, and sexual provocation. Asterisks denote statistically significant differences between sexes within the corresponding age group (* *p* < 0.05; ** *p* < 0.01; independent samples *t*-test).

To explore potential sex-based disparities in sexual offense victimization, differences in rates were analyzed across six age groups (0–17, 18–25, 26–40, 41–50, 51–65, and >65 years) (Table 1 and Figure 5). Overall, women exhibited significantly higher victimization rates than men across most offense types and age groups. The largest gaps were observed in adolescence, particularly in grooming, where rates among girls under 18 reached 172.0 per 100,000 compared to 69.3 in boys (*p* < 0.001), and in corruption of minors, with 110.3 per 100,000 in females versus 67.4 in males (*p* < 0.05). Sexual harassment also showed a pronounced imbalance, with adolescent females reporting 48.5 per 100,000 versus 14.1 in males (*p* < 0.01). The disparity persisted in adulthood, although the magnitude varied by offense type. In sexual abuse, women recorded significantly higher rates than men in the 18–25 (61.2 vs. 35.6 per 100,000; *p* < 0.05) and 26–40 age groups (54.7 vs. 22.3; *p* < 0.001). Sexual harassment displayed significant sex differences across all adult stages, peaking in the 51–65 age group, where women reached 22.8 per 100,000 compared to almost zero in men (*p* < 0.001). Other offenses showed sex gaps concentrated in specific adult life stages. In child sexual abuse images, female rates exceeded those of men in the 26–40 age group (15.2 vs. 6.4 per 100,000; *p* < 0.05). Exhibitionism presented significant differences in the 18–25 group (10.7 vs. 3.1; *p* < 0.05), while sexual provocation peaked among minors, with women showing nearly double the rate of men (21.5 vs. 11.2 per 100,000; *p* < 0.05).

**Figure 6:**
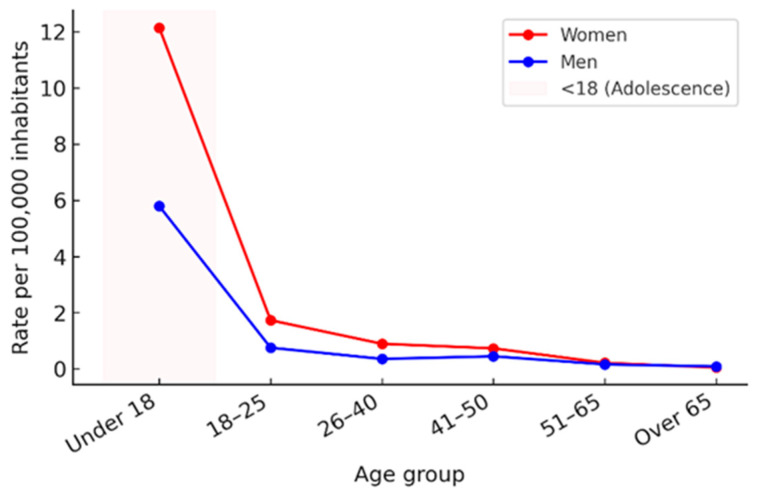
Mean cybersexual victimization rates by age group, Spain 2011–2022. Mean rates per 100,000 inhabitants for women (red) and men (blue) are shown across six age groups. Statistically significant sex differences (*t*-test, *p* < 0.05) were observed in the groups under 18, 18–25, 26–40, and 41–50 years, while no significant differences were found in the 51–65 and over-65 categories. The shaded area highlights the “Under 18” group, representing adolescence according to the classification used in the dataset.

Figure 6 shows the comparison of mean cybersexual victimization rates per 100,000 inhabitants between women and men across age groups. *T*-test results revealed significant differences in most age categories: under 18 years (*p* = 0.001), 18–25 years (*p* = 0.005), 26–40 years (*p* < 0.001), and 41–50 years (*p* = 0.025). In contrast, no statistically significant differences were observed for the 51–65 (*p* = 0.185) and over-65 groups (*p* = 0.113). These findings indicate that gender disparities in cybersexual victimization are most evident during adolescence and early to middle adulthood, while rates converge in later life stages.

### 3.6. Projected Trends in Cybersexual Victimization Rates in Spain by Sex and Age Group (2011–2035)

**Figure 7:**
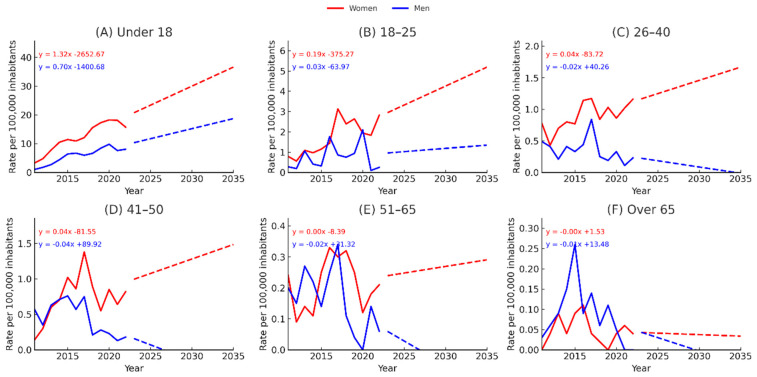
Projected trends in cybersexual victimization rates in Spain by sex and age group (2011–2035). Rates of cybersexual victimization per 100,000 inhabitants are shown for women (red) and men (blue) across six age groups (**A**–**F**). Solid lines represent observed data (2011–2022) and dashed lines represent linear projections up to 2035. Equations for the fitted linear trends are displayed in each panel, corresponding to the slope and intercept of the estimated rates. Projections indicate persistent gender disparities across all age groups, with the largest gap in adolescence (<18), where female rates are projected to remain nearly twice those of males.

As shown in Figure 7, projections of mean cybersexual victimization rates per 100,000 inhabitants up to 2035 confirm a persistent disparity between women and men across all age groups. The gap is particularly marked among those under 18 years old, where the projected female trajectory follows the equation y = 1.32x − 2652.67, compared to y = 0.70x − 1400.68 for men. These results indicate that victimization among girls under 18 is expected to remain substantially higher than among boys, with both groups increasing but at a sharper rate for females. In the 18–25 age group, women are projected to increase according to y = 0.19x − 375.27, while men follow a much flatter trend (y = 0.03x − 63.97). Although absolute rates are lower than in minors, the female curve remains consistently above the male one throughout the period. The projections for individuals aged 26–40 reveal continued female predominance, with women following y = 0.04x − 83.72 against a slightly declining male trend (y = −0.02x + 40.26). A similar pattern emerges in the 41–50 bracket: women show an increasing trajectory (y = 0.04x − 81.55), whereas men remain nearly flat or decreasing (y = −0.04x + 89.92). These age groups suggest that the female excess in cybersexual victimization risk is not confined to adolescence but extends into adulthood. The relative disparity is also evident among those aged 51–65: male rates remain close to zero (y = −0.02x + 31.32), while women display a slight upward trend (y = 0.00x − 8.39). Finally, in the over-65 category, absolute rates are lower, but the gender gap persists, with women projected at y = −0.00x + 1.53 and men at y = −0.01x + 13.48.

## 4. Discussion

The aim was to analyze sex- and age-specific temporal trends and projections of cybersexual victimization in Spain (2011–2022), disaggregated by sex, age group, autonomous community, and offense type, to identify where disparities emerge and persist (particularly from adolescence (<18) into midlife) while also examining gender and regional inequalities to provide evidence for prevention strategies that are both gender-sensitive and tailored to different developmental stages and territorial contexts.

Existing research shows that cyber-sexual crimes are widespread and strongly influenced by structural inequalities (Henry et al., 2020; Henry & Powell, 2016). Evidence concerning children and adolescents is particularly alarming. In the United States, a nationally representative survey found that, before the age of 18, 15.6% of respondents had experienced online child sexual abuse, 11.0% image-based abuse, 5.4% grooming, and 3.5% sextortion (Finkelhor et al., 2022). In the United Kingdom, reports indicate that 39% of children aged 8–17 have experienced bullying, primarily online, while official statistics show that 19.1% of children aged 10–15 experienced online bullying in the past year (Flatley, 2017). In Spain, recent survey data indicate that 60.6% of young people have experienced some form of digital sexual violence, with the most frequent behaviors being the non-consensual receipt of sexual content (22.1%) and harassment based on physical appearance (21.3%) (Calderón Gómez et al., 2024). These figures highlight the urgent need for studies that combine police data with self-reported victimization surveys, as only this approach can provide a comprehensive picture of the magnitude and patterns of cybersexual victimization.

Our results showed a sustained increase in cybersexual offenses, particularly in grooming, child sexual abuse images, and contact crimes. These offenses demonstrated sharp growth throughout the study period and exhibit worrisome projections through 2035, underscoring the urgency of preventive interventions. The projection analysis should be interpreted as a descriptive continuation of the empirical trends observed between 2011 and 2022, rather than as a predictive model of future incidence. The linear approach was adopted to provide a transparent, comparable, and replicable framework across all categories and demographic strata, thereby identifying where gender and age disparities persist or intensify if current patterns remain unchanged. Confidence intervals were intended to convey uncertainty, recognizing that reliability decreases progressively with temporal distance from the observed data. It is worth emphasizing that the temporal and projection analyses were conceived as descriptive tools. Their primary purpose was to quantify the velocity of change and to enable comparative interpretation between offense types, sexes, and age groups. Thus, rather than aiming to model causation or predict future crime rates, the regression results highlight where disparities are accelerating, stabilizing, or remaining constant over time. Grooming displayed the steepest upward trajectory, steadily increasing rates across the decade. Contact offenses, nearly non-existent in 2011, reached 0.9 per 100,000 in 2022, reflecting an emerging pattern of digitally facilitated predatory behavior that may indicate increased perpetration and improved reporting mechanisms. This escalation likely reflects the pervasive role of social media and private messaging platforms in enabling repeated and often anonymous contact between offenders and potential victims. The ease of access, low risk of detection, and persistent exposure to sexualized interactions may contribute to an environment where aggression is more likely to occur (Kim et al., 2023). From a behavioral standpoint, these patterns are consistent with the General Aggression Model, which highlights how online anonymity, reduced accountability, and exposure to sexualized content may weaken inhibitory control and increase the risk of aggressive sexual behavior (Anderson & Bushman, 2002). Child sexual abuse images exhibited the highest mean incidence across all offense categories and affected a broader age range than expected. Although minors were frequent victims, a substantial proportion of adult victims (particularly those aged 26 to 40) were also identified, suggesting diverse offender profiles and victimization pathways. These findings emphasize distinguishing between offenses involving direct victimization and those related to producing, possessing, or distributing illegal material (Endrass et al., 2009; Malamuth, 2018).

Marked regional disparities emerged, with the Balearic and Canary Islands and Andalusia consistently reporting higher rates of cybersexual offenses. In contrast, Galicia, Catalonia, and Castilla-La Mancha recorded lower incidence levels. These variations likely reflect structural asymmetries in digital access, education, and income distribution. Sexual abuse and corruption of minors were more frequent in socioeconomically disadvantaged areas, while grooming and child sexual abuse images were more prevalent in territories with higher digital penetration, suggesting distinct regional risk profiles. Interestingly, internet usage frequency did not directly correlate with specific offense types, indicating that mere access is insufficient to predict risk; instead, digital interaction quality, supervision, and context shape vulnerability (Helsper, 2017; Mee et al., 2024). It is important to acknowledge that the data’s regional distribution refers to where the victim filed the complaint, rather than the origin of the perpetrator. Therefore, the geographical scope of perpetrator activity cannot be determined from these records, while the location of victim reports remains a reliable indicator of where the crime was formally registered. Age- and sex-disaggregated data confirmed that female minors experienced the highest mean victimization rates, particularly in grooming, sexual abuse, and sexual provocation. Elevated rates among young women aged 18 to 25 (especially in harassment and contact offenses) suggest that digital sexual violence is not confined to adolescence but extends into early adulthood. These findings support the need for prevention strategies that are both gender-sensitive and developmentally targeted (Finkelhor et al., 2022; Lauritsen & Davis Quinet, 1995).

Higher incidence rates were concentrated in regions with lower educational attainment and greater socioeconomic vulnerability. Specifically, the regions with the highest incidence rates included Andalusia, Extremadura, and Murcia, followed by Castilla-La Mancha and the Canary Islands, among the Spanish territories with the lowest average income levels and the highest structural vulnerability. These findings reinforce the notion that structural disadvantages (such as poverty, limited digital literacy, and weak institutional safeguards) exacerbate the risk of online sexual victimization. Addressing these root causes is essential for developing equitable and effective cyberviolence prevention strategies (Bartolomei & Cava, 2024; Ceia et al., 2021; Odudu, 2024). The results of projections strengthen the evidence that cybersexual victimization in Spain is not only a current and markedly gendered phenomenon but one that is expected to persist and, in some age groups, intensify in the coming decade. The sustained and, in some instances, widening disparities between female and male victimization rates (most notably among those under 18) highlight the enduring vulnerability of adolescent girls and the persistence of elevated risk well into adulthood. Notably, projected rates for women remain consistently higher across all age groups, including midlife and older adulthood, indicating that preventive strategies cannot be confined to early adolescence. Instead, these findings call for a life-course approach that integrates gender-sensitive measures from early adolescence through later life stages, addressing the initial onset of vulnerability and its reinforcement through adult digital interactions.

A substantial proportion of cybersexual crimes, especially those involving minors or intimate contexts, likely remain unreported due to stigma, fear of retaliation, or institutional distrust (Pashang et al., 2018; Ray & Henry, 2025). Consequently, the actual scale of the phenomenon may be underestimated. The reliance on aggregated data also limited the analysis of individual or household-level risk factors, such as platform-specific exposure, online routines, or prior victimization history. Future research should integrate micro-level data to better understand how personal circumstances interact with structural risk determinants (European Institute for Gender Equality, 2017; Pérez & Sitges Maciá, 2025).

The predominance of female victims, particularly among minors and young adults, may reflect both genuine vulnerability and reporting asymmetries. Previous studies suggest that women may be more likely to recognize and report sexual harm online, while male victims may remain invisible due to stigma, underrecognition, or fear of disbelief. The gender gap in reported victimization may be partly shaped by differential thresholds for disclosure and access to support networks (Pashang et al., 2018; Ray & Henry, 2025).

Our results revealed consistent and statistically significant sex-based disparities in cybersexual victimization rates across most offense types and age groups, particularly among women. Victimization rates were significantly higher among females in offenses such as sexual harassment, corruption of minors, and sexual abuse, with these differences especially marked in younger age groups, notably those under 18 and between 26 and 50 years. These patterns align with previous research highlighting the heightened vulnerability of women and girls to online sexual exploitation and gender-based digital violence (Backe et al., 2018; Mas’udah et al., 2024; Sarkar & Rajan, 2023). Furthermore, offenses such as grooming and child sexual abuse images also showed significant differences in specific adult groups, with women experiencing notably higher rates, particularly in the 26–40 and 51–65 age brackets. These findings align with previous research indicating a connection between the consumption of child sexual abuse images and an increased risk of contact sexual abuse (Long et al., 2013), as well as with arguments that pornography functions as a structural driver of sexual violence and exploitation (Taylor, 2018).

In this context, the significantly higher rates of grooming and child sexual abuse image victimization observed among adult women, particularly in the 26–40 and 51–65 age groups, should not be viewed as statistical anomalies but rather as manifestations of broader gendered dynamics. These dynamics shape digital environments and reinforce patterns of vulnerability and exploitation that affect not only minors but also adult women in increasingly complex and pervasive ways.

Notably, sexual provocation and exhibitionism displayed fewer consistent sex-based differences, with only isolated adult groups showing statistically significant disparities. These results underscore the need for gender-sensitive approaches in both policy and prevention strategies targeting online sexual crimes (Marganski, 2020). The persistence of significant gaps across multiple offense types and age groups suggests that structural inequalities in digital environments continue to place women, especially younger women, at greater risk (Acilar & Sæbø, 2023; MacAya et al., 2021). From a preventive standpoint, these findings provide actionable insights for designing protection strategies adapted to different female age groups’ digital behaviors and vulnerabilities. The disproportionate increase in grooming and online sexual harassment among adolescents and young adult women calls for interventions that combine education on digital safety, early detection mechanisms, and gender-sensitive public awareness campaigns.

In Spain, several initiatives already provide a framework for the prevention and early detection of online sexual abuse. The national program “Internet Segura for Kids (IS4K)”, coordinated by the National Cybersecurity Institute (INCIBE) (Internet Segura for Kids (IS4K) | INCIBE, n.d.), promotes digital safety among minors through educational materials, awareness campaigns, and helplines for children, families, and educators. Likewise, the “IV Plan de Acción contra la Explotación Sexual Infantil y Adolescente (2021–2024)” (IV Plan de Acción contra la Explotación Sexual Infantil y Adolescente en España (2021–2024); Bienestar y Protección Infantil, 2020), endorsed by the Spanish Government and regional authorities, includes preventive and protective measures aimed at reducing sexual exploitation and abuse through inter-institutional coordination and professional training. These existing structures offer valuable platforms to strengthen gender- and age-sensitive interventions addressing the increasing prevalence of grooming and online sexual harassment among female adolescents and young women. Integrating the empirical trends identified in this study into these national strategies could enhance prevention, improve detection, and guide the design of more targeted protection programs across educational and community settings. Likewise, preventive policies should consider how structural inequalities (social, technological, and economic) shape women’s exposure to cybersexual victimization throughout their life course. Thus, the results of this study offer a valuable empirical foundation for targeted protection and prevention strategies in Spain. Finally, future research should adopt a more theory-driven approach to better explain the persistent gendered patterns observed in cybersexual victimization. Integrating theoretical frameworks that address socialization processes, gender power dynamics, and digital behavior could help clarify the mechanisms sustaining women’s and girls’ vulnerability in online environments and inform the development of more effective preventive and policy responses.

Future research should build on these findings by adopting longitudinal and mixed-method designs to better understand the temporal dynamics and causal pathways of cybersexual victimization. Further studies are needed to examine how structural inequalities, digital literacy, and socialization processes interact to shape vulnerability to online sexual abuse across the life course. Comparative and cross-national research could also help identify contextual factors that modulate risk and protection. At the same time, intervention studies assessing the effectiveness of prevention and awareness programs (such as IS4K or the National Action Plan against Child and Adolescent Sexual Exploitation) would provide valuable evidence to guide policy implementation. Integrating these complementary perspectives will be essential to developing a deeper and more holistic understanding of cybersexual victimization and its prevention.

The main limitation of this study was its reliance on police-reported data, which only reflects offenses that were formally reported to law enforcement. These data exclude unreported cases, which may represent a substantial portion of actual victimizations, and should be interpreted as patterns of reported victimization rather than as direct measures of overall prevalence. The observed increase in reported offenses may reflect both a real rise in abuse and greater willingness to report, facilitated by reduced stigma and more accessible reporting mechanisms. It should also be acknowledged that when a perpetrator offends multiple victims, each filed complaint is recorded separately, and in cases of multiple forms of victimization, only the specific offense reported is registered. This reporting structure may contribute to underestimating the number of incidents, but it still provides a reliable and consistent basis for analyzing long-term temporal and demographic patterns. Additionally, some autonomous communities lacked complete data in the early years (Catalonia and the Basque Country before 2015), which may have affected longitudinal comparability. A further limitation of this study is its reliance on police data, which only capture reported cases and inevitably underestimate the true prevalence of cybersexual victimization. To address this gap, future research should be designed to go beyond official records by incorporating self-reported victimization surveys. Combining both sources of information would allow for a more accurate and comprehensive understanding of the scope and dynamics of these offenses. The regional classification used in this study reflects the administrative organization of Spain and how police forces record and publish complaints. Although it is assumed that victims usually file reports in the place where the cybercrime occurred or where they experienced its effects, the precise location of the perpetrator or the digital act itself cannot be determined.

Nevertheless, the regional distribution of complaints provides valuable insight into territorial patterns of reported victimization. Another limitation concerns age grouping: while the World Health Organization (WHO) defines adolescence as the period from 10 to 19 years (subdivided into early and late adolescence), our dataset grouped victims into broader categories (<18 years, 18–25 years, etc.). Therefore, direct comparisons with research based strictly on WHO-defined age ranges should be made cautiously. A further methodological limitation concerns the use of simple linear regression for temporal projections. While this approach allowed for consistent and interpretable comparisons across offense types and demographic strata, it does not account for stochastic variation or temporal dependence. Future research could build upon these findings by incorporating more advanced time-series techniques (e.g., ARIMA, VAR, or Bayesian models) to explore potential non-linearities and random fluctuations with greater precision.

These findings call for developing behaviorally informed and structurally grounded prevention strategies, tailored to the distinct risk profiles of girls and young women. Interventions should prioritize the creation of safer digital ecosystems, early identification of risk patterns, and targeted education and reporting mechanisms, particularly in regions with compounded vulnerabilities. Future efforts should also address the institutional and cultural barriers that hinder disclosure and access to support, ensuring that preventive actions are not only reactive but also transformative.

## 5. Conclusions

This study demonstrated that cybersexual victimization in Spain disproportionately affects women across the life course, with the most pronounced disparities emerging before the age of 18, particularly in grooming and corruption of minors. Significant gender gaps persist into young adulthood (18–25 years) and midlife (26–40 and 41–50 years), especially in sexual harassment, sexual abuse, and sexual provocation. The elevated female victimization observed from adolescence into midlife decreases only slightly in older age groups, indicating that gendered vulnerabilities in digital environments are not confined to youth. These findings underscore the need for prevention strategies that address early onset of risk and its persistence across adulthood, ensuring that interventions are both developmentally targeted and gender-sensitive.

## Figures and Tables

**Table 1:** Sex-based comparison of cybersexual victimization rates in Spain by offense type and age group (2011–2022).

Offense Types	Age Groups	Mean (Men)	Mean (Women)	*t*	*p*-Value
Sexual abuse	Under 18	0.858	1.709	−3.21	**0.004**
	18–25	0.147	0.360	−2.85	**0.008**
	26–40	0.026	0.113	−2.95	**0.007**
	41–50	0.021	0.050	−2.50	**0.018**
	51–65	0.007	0.018	−2.10	**0.041**
	Over 65	0.003	0.003	0.00	1.000
Sexual harassment	Under 18	0.143	0.814	−3.80	**0.002**
	18–25	0.100	0.719	−3.45	**0.004**
	26–40	0.033	0.444	−3.15	**0.006**
	41–50	0.110	0.288	−2.40	**0.022**
	51–65	0.010	0.094	−2.25	**0.030**
	Over 65	0.002	0.012	−2.10	**0.041**
Corruption of minors	Under 18	1.584	2.741	−4.20	**0.001**
	18–25	0.155	0.238	−1.50	0.150
	26–40	0.063	0.073	−0.80	0.430
	41–50	0.072	0.089	−0.70	0.490
	51–65	0.024	0.028	−0.50	0.600
	Over 65	0.026	0.014	1.20	0.240
Grooming	Under 18	1.635	4.296	−4.60	**0.001**
	18–25	0.047	0.081	−1.10	0.280
	26–40	0.034	0.074	−1.50	0.150
	41–50	0.070	0.131	−1.60	0.140
	51–65	0.027	0.011	1.30	0.210
	Over 65	0.007	0.004	0.60	0.560
Exhibitionism	Under 18	0.207	0.551	−3.00	**0.009**
	18–25	0.004	0.000	1.00	0.320
	26–40	0.008	0.028	−1.20	0.240
	41–50	0.021	0.007	1.00	0.320
	51–65	0.002	0.000	1.00	0.320
	Over 65	0.007	0.002	0.90	0.350
Child sexual abuse images	Under 18	0.849	1.247	−2.70	**0.012**
	18–25	0.256	0.270	−0.30	0.770
	26–40	0.179	0.124	1.10	0.280
	41–50	0.207	0.135	1.20	0.240
	51–65	0.085	0.050	1.00	0.320
	Over 65	0.036	0.009	1.50	0.150
Sexual provocation	Under 18	0.519	0.787	−2.20	**0.036**
	18–25	0.043	0.062	−1.10	0.280
	26–40	0.010	0.034	−1.50	0.150
	41–50	0.028	0.029	−0.10	0.910
	51–65	0.006	0.011	−0.80	0.430
	Over 65	0.005	0.004	0.20	0.840

Mean victimization rates per 100,000 inhabitants are presented for men and women across six age groups. Independent samples *t*-tests were performed to assess statistical significance. Statistically significant differences (*p* < 0.05) are in bold.

## Data Availability

The data presented in this study are openly available at https://estadisticasdecriminalidad.ses.mir.es/publico/portalestadistico/publicaciones.html (accessed on 13 January 2025).
